# Lithium Prescribing on an Older Adult Inpatient Ward and Trialling Improvements to Communication on Discharge

**DOI:** 10.1192/bjo.2024.615

**Published:** 2024-08-01

**Authors:** Wishwanath Patkee, James Barclay

**Affiliations:** Hertfordshire Partnership NHS Foundation Trust, Hatfield, United Kingdom

## Abstract

**Aims:**

Lithium is the recommended first-line pharmacological treatment for bipolar disorder and as an augmentation of the treatment for depression. Both NICE and local guidelines stipulate the need for patient counselling regarding side effects, interactions and toxicity, alongside strict monitoring requirements for initiation and maintenance.

We aimed to assess compliance with these guidelines for patients prescribed lithium on a functional older adult inpatient ward in Hertfordshire Partnership NHS Foundation Trust (HPFT). Additionally, following feedback from the local crisis and community colleagues, concerns were emphasised around inconsistent communication on discharge. We therefore also aimed to evaluate the introduction of a small-scale intervention to the method of discharge communication.

**Methods:**

A retrospective analysis of electronic patient records was undertaken for the 43 patients within HPFT prescribed lithium during their inpatient stay on a functional older adult ward over a five-year period (2019–2023).

Lithium monitoring on drug initiation was assessed for compliance with the standards set by NICE guidelines for the management of bipolar disorder. For all patients prescribed lithium, we also noted demographics, diagnosis, rate of side effects and toxicity, discontinuation, and documentation of discharge communication to the community. A standardised template for communication with community and crisis colleagues was introduced, and its impact was assessed.

**Results:**

58% (n = 25) of patients were initiated on lithium, with 80% (n = 20) of them having documentation of counselling. Baseline blood tests were consistently recorded for all newly prescribed lithium patients (n = 25), and regular serum monitoring was present in all patients. Common side effects included tremors (26%; n = 11) and polyuria (7%; n = 3), while in 63% of patients (n = 27), no side effects were noted. Toxicity occurred in four cases, leading to discontinuation in 50% of them.

Prior to concerns being highlighted around handovers to community colleagues, there was specific documentation of a handover in 19% (n = 6) of cases. Following consultation with stakeholders and consensus regarding the trial of a template for communication to the patient's community consultant, documentation improved to 75% (n = 6).

**Conclusion:**

All patients in this study who were initiated or maintained on lithium received serum monitoring as inpatients in accordance with NICE guidelines. The introduction of small-scale improvements with a standardised template has been effective, significantly improving discharge communication with community colleagues for patients on lithium. Further research is necessary to elucidate the impact of these changes on patient care in the community by gathering feedback from a diverse group of community colleagues.

